# Design and study of psychometric properties of the Community Attitude to Abortion Scale (CAAS) with the Chilean population: Autonomy and Stigma

**DOI:** 10.3389/fpsyg.2022.1008492

**Published:** 2022-12-23

**Authors:** Beatriz Pérez Sánchez, Juan José Burgos Padilla, Carolina Alveal-Álamos, Andrés Concha-Salgado, Luisa Jara Sepúlveda, Francisco Javier Rodríguez Díaz

**Affiliations:** ^1^Department of Psychology, University of La Frontera, Temuco, Chile; ^2^Department of Psychology, University of Oviedo, Oviedo, Spain; ^3^Nucleus in Social Sciences and Humanities, University of La Frontera, Temuco, Spain

**Keywords:** attitudes, abortion, scale, psychometric properties, autonomy, stigma, religiosity, political orientation

## Abstract

**Introduction:**

Attitudes toward abortion are related to structural, cultural, and direct gender-based violence. This violence can affect women’s mental, physical and reproductive health. Therefore, it is essential to know the nature of community attitudes toward abortion. Since we currently do not have an instrument that measures attitudes towards abortion in Chile, we set the objective of this study to design the *Community Attitude to Abortion Scale (CAAS)* and analyze its psychometric properties in a Chilean community population.

**Methods:**

This work is an instrumental design study. Using a sampling of panelists by sociodemographic quotas, we obtained a sample of 1,223 participants with a mean age of 36.7 years (*SD* = 13.56).

**Results:**

As a result, we obtained a scale of 18 items and two correlated factors, *Autonomy* and *Stigma*. This structure fits better as an Exploratory Structural Equations Model (ESEM). Both factors have excellent internal consistency. In addition, we obtained evidence of concurrent and discriminant validity: The scores on the factors of the Universal Religious Involvement Scale (I-E12) correlated negatively with *Autonomy* and positively with *Stigma*; participants with low levels of identification with a right-wing political orientation, with high levels of identification with a leftwing, pro-feminist, pro-LGBTQ +, and pro-euthanasia political orientation, obtained higher mean scores on *Autonomy* and lower on *Stigma*.

**Discussion:**

The CAAS is an adequate tool for use with the Chilean community population, with evidence of consistency and validity. La CAAS is the first tool to measure attitudes to abortion in this country.

## Introduction

Attitudes toward abortion are a relevant construct to understand opinion trends, violence against women in the framework of reproductive rights, and the legislative changes that have occurred on this matter in recent years in various countries. This is the case in Chile, where abortion has recently been legalized in some circumstances, after decades of prohibition and with varying acceptance levels. However, the number of instruments that measure this construct with adequate psychometric properties is scarce. Furthermore, none of them has been adapted with success or designed for the Chilean population. For this reason, we set the objective of this research to design the *Community Attitude to Abortion Scale (CAAS)* and analyze its psychometric properties in the Chilean community.

Attitudes towards abortion are conceptualized as a lasting organization of beliefs and cognitions endowed with an affective charge. This affective charge can be in favor or against the voluntary interruption of pregnancy (VIP) and predisposes to actions consistent with said cognitions and affects (Festinger, 1964). According to the Theory of the Triangle of Violence (Galtung, 1990), the expression of negative attitudes as a form of gender violence is exerted through three closely related dimensions: structural, cultural, and direct. This conceptualization is consistent with the Ecological Models of Abortion Stigma (Kumar et al., 2009) and the proposal of the Bellagio group on the levels at which it operates this stigma (Hessini, 2014).

### Structural violence against abortion

Negative attitudes towards abortion are associated with less agreement with policies supporting access to abortion (Patev et al., 2019a; Cutler et al., 2021). From a structural perspective, we find very diverse forms of legislation against reproductive rights at the international level, with the strictest restrictions being classified as a violation of human rights (Human Rights Committee, 2018). Chile is positioned as one of the Latin American countries with a more restrictive legislative tradition (Ramos, 2016; Dides-Castillo and Fernández, 2018; Maira et al., 2019). Except between 1931 and 1989, years in which therapeutic abortion was legal, abortion has been considered a crime under any circumstance from 1874 (Donoso and Vera, 2016; Osorio, 2022) until 2017, the year in which Law 21,030 re-decreed the legality of abortion for three causes: (Festinger, 1964) risk of death for the woman; (Galtung, 1990) lethal fetal in viability; and (Kumar et al., 2009) violation (Ministerio de Salud, 2017). However, other forms of structural violence derive from this Law. For example, the institutional conscientious objection, unequal access to abortion services depending on the place of residence or socioeconomic level, lack of information regarding the Law to guarantee its access, and insufficient training and participation of the personnel regarding the VIP (Frez, 2018; Robledo, 2018; Marshall and Zúñiga, 2020).

### Cultural violence against abortion

At a cultural level, negative attitudes towards VIP are based on beliefs, values, and social norms of a traditional and conservative nature—for example, the value of responsibility and care for others over self-determination and the archetypes of femininity (female sexuality only for reproduction, the inevitability of motherhood, and the instinctive care of children). Another example is the defense of respect for the right to life from conception to natural death (Bègue, 2001; Kumar et al., 2009; Vitti and Cabello, 2010; Norris et al., 2011; Piazza, 2012; Clements, 2014; Adesse et al., 2016; Pfeffer, 2017; Prusaczyk and Hodson, 2018). These values outline VIP as selfish behavior, which transgresses the essential nature of women, and even as murder, leading to the stereotyped characterization of women who abort as unintelligent, inferior, sinful, dirty, unreliable, incomplete, and promiscuous (Shellenberg et al., 2014; Sorhaindo et al., 2014; Adesse et al., 2016). In Chile, people oppose abortion, describing the woman who interrupts her pregnancy as cold, insensitive, irresponsible, and selfish (Pérez et al., 2020).

These beliefs, values, and social norms can vary in intensity and content not only between individuals but also between social groups and sociocultural settings or countries. Consistent with this, also the conceptualization and expression of attitudes and stigma toward abortion (Kumar et al., 2009; Hanschmidt et al., 2016). At the group level, these values and beliefs are part of the foundation of religious doctrines and right-wing political orientation. Both are social identities with significant weight in forming personal identity and correlate of greater importance in explaining attitudes towards abortion (Bahr and Marcos, 2003; Hendriks, 2012; Lizotte, 2015; Patev et al., 2019a,b; Pérez et al., 2020; Cutler et al., 2021; Osborne et al., 2022; Pérez et al., 2022). Thus, those who identify with these groups often question other identities, rights, or individual freedoms closely related to these values (Hessini, 2014). For example, religiosity is associated with the rejection of sexual minorities, a relationship explained by authoritarianism and traditional beliefs about gender (Janssen and Scheepers, 2019); it is an essential indicator of the refusal of euthanasia, a practice that defies the religious mandate that only God can take life (Stets and Leik, 1993; Pfeffer, 2017; Francis et al., 2019); and it is also an antagonistic identity to feminism in gender issues, becoming an indicator of hostile sexism when both identities coexist in the same individual (Hernandez, 2021). In Chile, the position of religious groups against the VIP has been verified, exerting their power and influence on public opinion (Dides-Castillo and Fernández, 2018; Nicholls and Cuestas, 2018; Elgueta et al., 2019; Marshall and Zúñiga, 2020; Pérez et al., 2020, 2022).

We found differences in attitudes toward abortion between countries, according to variations of impact on society of conservative social groups and the beliefs, values, and social norms that support them. For example, Bahr and Marco (Bahr and Marcos, 2003) found differences between the Greek and American population according to the impact of religiosity on attitudes through sexual liberalism; Sahar and Karasawa (Sahar and Karasawa, 2005) found a greater influence of symbolic politics on attitudes towards abortion in the Japanese population compared to the American people. Mosley et al. (Mosley et al., 2020) conclude that attitudes towards abortion are related to each nation’s socioeconomic and gender ideology. These variations are also found in the legislative expression, considering the regulatory diversity of abortion between countries or specific beliefs. For example, in Ghana and Zambia, it is believed that the woman who aborts can spread diseases, a belief that is not installed in other sociocultural realities (Shellenberg et al., 2014).

### Direct violence against abortion

Finally, direct violence is expressed in treatment and concrete actions at the individual level. Kumar et al. (Kumar et al., 2009) point out that carrying the label of a woman who aborts causes her to be separated and considered part of an “other,” suffering a loss of status, rejection, exclusion, and discrimination. Those with negative attitudes believe VIP is a shameful action that should be kept out secretly, a sin that deserves punishment (McMurtrie et al., 2012; Hanschmidt et al., 2016), such as infertility (Sorhaindo et al., 2014). In addition, women are deserving of rejection by men and the rest of the community (Shellenberg et al., 2014; Sorhaindo et al., 2016).

We can point to precise acts of direct violence. For example, pro-life groups organize in front of abortion clinics to dissuade women with lies and encourage women and professionals to repent (Morgan, 2017; Lowe, 2019; Lowe and Page, 2019). The literature also shows that there is direct violence on the part of some health professionals. For example, through accusatory or prejudice-based comments, threats of denunciation, moral judgments or humiliating treatment as criminals or suspects, disclosure of medical history without consent, refusal to provide relief of pain or absence of analgesics, neglect and abandonment, and lack of support and containment (Jardim and Modena, 2018; Williams et al., 2018; Makleff et al., 2019).

Studies carried out with the Chilean population confirm that people against the VIP support the punitive treatment of abortion (Pérez et al., 2020); they threaten political women who speak out in favor of abortion on social networks and question their competence (Pérez-Arredondo and Graells-Garrido, 2021). On the other hand, health professionals accept conscientious objection alleging doubts about the credibility of women and demanding more significant participation of family and partner in decision-making for the VIP (Muñoz et al., 2021; Alveal-Álamos et al., 2022), exerting humiliating treatment on migrant or racialized women who want access to legal abortion (Osorio, 2022).

### The consequences of stigmatizing attitudes towards abortion

The consequences of this treatment impact women in various ways (Hanschmidt et al., 2016). Those who perceive themselves as stigmatized manifest mental health problems, such as depression, anxiety, stress, psychological distress, social withdrawal, avoidance behaviors, and somatic symptoms (American Psychological Association, 2008; O’Donnell et al., 2018; Moreno López et al., 2019). Added to this is that internalized stigma generates feelings of guilt and shame, factors that lead women to keep the practice of VIP a secret (Astbury-Ward et al., 2012; Sorhaindo et al., 2014), retract their decision (Ramos, 2016), or even expose themselves to unsafe (and illegal) methods to achieve it (McMurtrie et al., 2012; Mosley et al., 2017). The real figures on secretive abortions practiced each year are unknown in Chile. However, studies based on estimates and with indirect methodology predicted that by 2015 a total figure of close to 300,000 clandestine and unsafe abortions was reached (Dides-Castillo and Fernández, 2018).

In short, the scope of violence motivated by community attitudes towards abortion and its direct impact on women’s health and internalized stigma justifies the need to learn more about these attitudes. Focusing our attention on these attitudes allows us to focus on the cause of this problem.

### The measure of attitudes towards abortion

In order to know the community attitudes towards abortion, it is necessary to have instruments that have studies on their psychometric properties, which evaluate beliefs and cognitions of the community about abortion and women who have had an abortion, and under the current Chilean sociocultural scenario. In the literature, we located several instruments that could be adapted for use in Chile.

Self-report instruments exist to assess explicit attitudes towards abortion developed with populations from the USA, Australia, Ghana, Zambia, and Mexico (see Table 1). The first scales designed, the *Abortion-Attitude Scale* (Snegroff, 1976), the *Abortion Attitudes Scale* (Stets and Leik, 1993), and the *Attitudes about abortion Scale* (Hill, 2004), were created in the US with a university population. However, in other cultural realities, only the second (Snegroff, 1976; Hill, 2004) and the third (Martin et al., 2020) have been used recently. Nevertheless, both evaluate the level of agreement with abortion in a series of circumstances and not cognitions and beliefs. *Abortion-Providing Physicians Scale* (AAAPPS; Martin et al., 2020) is the fourth instrument developed in the US but to assess health professionals’ attitudes towards abortion providers.

**Table 1:** Scales that have been developed to measure attitudes towards abortion.

	**Instrument**	**Sample**	**Internal structure**	**Reliability**	**Evidence of validity based on the relationship with other variables**
**USA**					
1	Abortion-Attitude Scale (Snegroff, 1976)	N = 527 students Men = 266 Women = 261	30 items Likert scale (5 points) One dimensional	There is no information	Attitudes toward abortion correlate significantly and positively with knowledge about abortion
2	Abortion attitudes scale (Stets and Leik, 1993)	N = 309 students	20 items Likert scale (5 points) Factors: (Festinger, 1964) Availability; (Galtung, 1990) Moral acceptability; (Kumar et al., 2009) Women’s autonomy in the decision to abort	*Ω _F1_* = 0.96 *Ω _F2_* = 0.95 *Ω _F3_* = 0.73	According to the scores obtained in each factor, the pro-lifers are: (Festinger, 1964) politically conservative; (Galtung, 1990) religious; (Kumar et al., 2009) moral absolutists; and have a conservative view on (Hessini, 2014) euthanasia; (Cutler et al., 2021) prayer in schools; (Patev et al., 2019a) and birth control
3	Attitudes about Abortion Scale (Hill, 2004)	N = 63 female students Mean age = 18.86	10 items Likert scale (7 points) One dimensional	There is no information	There was no relationship between attitudes towards abortion and cognitive complexity
4	Attitudes About Abortion-Providing Physicians Scale (AAAPPS) (Martin et al., 2020)	N = 560 physicians; Men = 261 Women = 270 Over 25 years	24 items Five-point Likert-type scale Factors: (Festinger, 1964) opinion; (Galtung, 1990) motivation; and (Kumar et al., 2009) competition.	α *_F1_* = 0.95 *α _F2_* = 0.81 *α _F3_* = 0.80 *α _Total_* = 0.94	Favorable attitudes toward providers were inversely related to (Festinger, 1964) attendance at religious events; and positively with (Galtung, 1990) support for the legality of abortion; and (Kumar et al., 2009) the idea that abortion is important for women’s equality. Attitudes were more favorable among abortion providers: (Hessini, 2014) with children; and (Cutler et al., 2021) who had referred a patient for an abortion
**Australia**					
5	Adolescent Attitudes to Abortion Scale (AAA) (Hendriks, 2012)	N = 406; Men = 203 Women = 203 Between 12 and 19 years old	9 items (1 only for men and 1 only for women) Likert scale (4 points) One dimensional	*PSI* = 0.82	Attitudes were more favorable among adolescents: (Festinger, 1964) older; (Galtung, 1990) women, (Kumar et al., 2009) non-Aboriginal; (Hessini, 2014) non-religious; (Cutler et al., 2021) sexually active; (Patev et al., 2019a) and with previous pregnancy experience
**Ghana and Zambia**					
6	Stigmatizing Attitudes, Beliefs, and Actions Scale (SABAS) (Shellenberg et al., 2014)	N = 531; Men =258 Women = 273 Between 18 and 49 years old	18 items. Likert scale (4 points) Factors: (Festinger, 1964) Negative stereotypes; (Galtung, 1990) Discrimination/exclusion; (Kumar et al., 2009) Fear of contagion	α *_F1_* = 0.85 α *_F2_* = 0.80 α *_F3_* = 0.80 α *_Total_* = 0.90	Attitudes were more favorable among participants who support the legalization of abortion
**Ghana**					
7	Abortion as a Right Scale; Moral Objection to Abortion Scale (Rominski et al., 2017)	N = 1.038 students Men = 556 Women = 480	Abortion as a Right Scale 5 items Likert scale (5 points) One dimensional Moral Objection to Abortion Scale 3 items Likert scale (5 points) One dimensional	*α _F1_* = 0.76 *α _F2_* = 0.74	Participants score highest on abortion as a Right Scale, when: (Festinger, 1964) they are over 25 years old; (Galtung, 1990) have sexual experience; (Kumar et al., 2009) have a partner; (Hessini, 2014) or know someone who has had an abortion. None of the above variables was significantly related to the Moral Objection to Abortion Scale
**Mexico**					
8	Scale of Attitudes towards Legal Assisted Abortion (EAALA) (García, 2012)	N = 130 students; Men =25 Women =105 Between 18 to 29 years old	19 items. Likert scale (4 points) Factors: (Festinger, 1964) Moral ambivalence; (Galtung, 1990) Pragmatic ambivalence; (Kumar et al., 2009) Anti-abortion; (Hessini, 2014) Diversity	*α _Total_* = 0.60 Data by factors are not included	Not included
9	Community Level Abortion Stigma Scale (CLASS) (Sorhaindo et al., 2016)	N = 5.600 residents; Men = 2.688 Women = 2.912 Over 25 years	23 items. Likert scale (4 points) Factors: (Festinger, 1964) Autonomy; (Galtung, 1990) Discrimination; (Kumar et al., 2009) Religion; (Hessini, 2014) and Secret	*α _F1_* = 0.78 *α _F2_* = 0.87 *α _F3_* = 0.88 *α _F4_* = 0.82 α _Total_ = 0.87	They are more likely to report stigmatizing attitudes: (Festinger, 1964) older, less educated, and more religious when other observable characteristics are held constant; (Galtung, 1990) the religious; and those who do not live in the metropolitan area of Mexico City They do not influence: (Festinger, 1964) gender; (Galtung, 1990) employment status; (Kumar et al., 2009) political affiliation; (Hessini, 2014) marital status; (Cutler et al., 2021) and the number of children.
10	Induced Abortion Attitudes Questionnaire (CAAI) (Marván et al., 2018)	N = 764 students; Over 18 years	23 items. Likert scale (5 points) Factors: (Festinger, 1964) Pro-life; (Galtung, 1990) Pro-choice; and (Kumar et al., 2009) Reproductive Rights	*α _F1_* = 0.91 *α _F2_* = 0.90 *α _F3_* = 0.70	Not included

Hendriks et al. (Bahr and Marcos, 2003) developed the Adolescent Attitudes to Abortion Scale (AAA) with an Australian adolescent population. On the other hand, the *Stigmatizing Attitudes, Beliefs, and Actions Scale* (SABAS; Shellenberg et al., 2014) and *Abortion as a Right Scale*; *Moral Objection to Abortion Scale* (Rominski et al., 2017) were developed with a community sample of Ghana and university women in Ghana and Zambia, respectively. Among these, the most used subsequently is the SABAS. For example, by Patev et al. (2019a,b) with a US population, or by (Holcombe et al., 2018) with an Ethiopian population. However, SABAS is adjusted to a sociocultural reality far removed from the Chilean one.

Finally, Mexico is the only Latin American country in which scales have been developed for the evaluation of attitudes towards abortion, a sociocultural reality closer to the Chilean one: *Scale of Attitude towards Legal Assisted Abortion* (EAALA; García, 2012) with students college students; *Abortion Stigma Scale at the Community Level* (CLASS; Sorhaindo et al., 2016), with community population; and the *Questionnaire of Attitudes towards Induced Abortion* (CAAI; Marván et al., 2018), with university students. Of these, the CLASS presents a robust study for its development and has subsequently been used in the US (Cutler et al., 2021). However, the CLASS (Pérez et al., 2022) showed no adjustment in a Chilean community sample.

### Objectives and hypotheses

In Chile, there is structural, cultural, and direct violence against women who have had an abortion. Also, we have seen the potential consequences of this violence for women and the absence of an instrument about attitudes towards abortion adapted to the current Chilean sociocultural reality. Because of this, we set ourselves the general objective of this study, to design the *Community Attitudes to Abortion Scale* (CAAS) and analyze its psychometric properties in the Chilean community population. Once the construct to be measured has been delimited, a battery of items has been generated, its quality has been evaluated through expert judgment, and those items with good psychometric properties have been identified and selected, we set ourselves the following specific objectives: (Festinger, 1964) to descriptively analyze the items of the CAAS; (Galtung, 1990) demonstrate evidence of validity based on the internal structure of the CAAS; (Kumar et al., 2009) provide evidence of reliability by internal consistency of the CAAS; (Hessini, 2014) demonstrate evidence of validity of the CAAS based on the relationship with other theoretically related variables: religiosity; identification with a leftist political orientation; with a right-wing political orientation; pro-feminism, pro-LGBTIQ+, and pro-euthanasia.

As a hypothesis, we hope to obtain a parsimonious scale whose items have a high discriminative capacity (H1). In addition, considering the various constructs and dimensions of attitudes that the existing instruments in the literature have addressed, we hypothesize that this instrument will have a multidimensional structure (H2) and that it will have an internal consistency equal to or greater than 0.7 (H3). Assuming that a higher score on the scale indicates a greater presence of negative attitudes, we hypothesize that attitudes towards VIP will correlate positively with religiosity (H4a). In addition, with an effect size between intermediate and large, we expect that the participants who show less negative attitudes towards the VIP are people: with low levels of identification with a right-wing political orientation (H4b), with high levels of identification with a left-wing political orientation (H4c), pro-feminist (H4d) proLGBTIQ + (H4e) and pro-euthanasia (H4f).

## Materials and methods

### Design

This work is an instrumental design study (Ato et al., 2013) since it consists of the design and study of the psychometric properties of a scale. For the selection of evidence of validity and reliability and selection of statistical analyses, we consider the methodological recommendations of Abad et al. (2011).

### Participants

The sample consisted of 1,223 participants with a mean age of 36.7 years (SD = 13.56), close to the country’s population mean age of 35.8 years (Instituto Nacional de Estadísticas, 2018). We used a sampling of panelists by sociodemographic quotas. We considered the geographic macrozone (15% from the north, 60% from the center, and 25% from the south of the country) according to the density distribution—population (Instituto Nacional de Estadísticas, 2018). In addition, we seek a balanced representation in the total sample based on gender (50% men and 50% women), age (50% between 18 and 30 years, and 50% from 31 years onwards), and socioeconomic level, following indications of the classification system of the Association of Market Researchers (33.3% high level-AB, C1a and C1b-; 33% medium level-CA and C3-; and 33.3% low level-D and E -). The inclusion criteria were to be Chilean and older than 18 years old (see Table 2).

**Table 2:** Descriptive data of the total sample and stratified by country zone.

		**Country zone**			**Total**
		**North (*n* = 182; 14.9%) *n* (%)**	**Center (*n* = 735; 60.1%) *n* (%)**	**South (*n* = 306; 25%) *n* (%)**	
Gender	Men	109 (59.9)	362 (49.3)	148 (48.4)	619 (50.6)
	Woman	73 (40.1)	373 (50.7)	158 (51.6)	604 (49.4)
Age	From 18 to 30 years	39 (21.4)	381 (51.8)	186 (60.8)	606 (49.6)
	31 years or older	143 (78.6)	354 (48.2)	120 (39.2)	617 (50.4)
Social class	Lower	53 (29.1)	226 (30.7)	124 (40.5)	403 (33)
	Middle	48 (26.4)	275 (37.4)	87 (28.4)	410 (33.5)
	High	81 (44.5)	234 (31.8)	95 (31)	410 (33.5)
Education level	Middle or lower	43 (23.6)	169 (23)	77 (25.2)	289 (23.6)
	Technique incomplete	10 (5.5)	55 (7.5)	19 (6.2)	84 (6.9)
	Technique Complete / Univ. incomplete	41 (22.5)	216 (29.4)	89 (29.1)	346 (28.3)
	University complete or Postgraduate	88 (48.4)	295 (40.1)	121 (39.5)	504 (41.2)
Native people	Nope	133 (73.1)	581 (79)	231 (75.5)	945 (77.3)
	Mapuche	11 (6)	130 (17.7)	71 (23.2)	212 (17.3)
	Other	38 (20.9)	24 (3.3)	4 (1.3)	66 (5.4)
Marital status	Single	59 (32.4)	393 (53.5)	168 (54.9)	620 (50.7)
	Married	99 (54.4)	277 (37.7)	120 (39.2)	496 (40.6)
	Separated, Divorced, or Widowed	23 (12.6)	64 (8.7)	18 (5.9)	106 (8.7)
Zone	Rural	14 (7.7)	57 (7.8)	23 (7.5)	94 (7.7)
	urban	168 (92.3)	678 (92.2)	283 (92.5)	1,129 (92.3)

### Instruments

#### Sociodemographic questionnaire *ad hoc*

This instrument collected information on sociodemographic aspects, such as age, gender, or educational level, and identification with social groups based on five 5-point Likert-type items, where 1 = *strongly disagree* and 5 = *strongly agree*. The five items began as follows, “I have a lot in common with the person…,” and ended by pointing to different social groups: (Festinger, 1964) average left-wing political orientation; (Galtung, 1990) average right-wing political orientation; (Kumar et al., 2009) feminist average; (Hessini, 2014) average defender of LGBTIQ+ rights; (Cutler et al., 2021) average advocate of euthanasia.

#### Community Attitude to Abortion Scale (CAAS)

We define the theoretical construct of attitudes towards abortion as global and relatively stable evaluations of the VIP and the woman who decides to have an abortion at some point in her life, positive or negative, and at a cognitive, affective, and/or behavioral level. In addition, we elaborated an initial battery of 97 items distributed in six theoretical dimensions (The prime of your life, Positive Stereotypes, Entitlement, Negative Stereotypes, Discrimination, and Morality) through (Festinger, 1964) a review of the existing scales in the scientific literature (see Table 1). Galtung (1990) analysis of interviews used in a previous study (Pérez et al., 2020) on value arguments about the VIP with the Chilean community population; and Kumar et al. (2009) analysis of social representations about the VIP through a discussion group with 6 Chilean activists in favor of free abortion.

Next, we conducted an expert consultation with 11 professionals from areas related to the subject of study *via* email to evaluate the conceptual, linguistic, and cultural relevance of the definition of the theoretical construct, its dimensions, and the initial battery of 97 items. As a result, the description of the theoretical construct and dimensions is maintained; eight items were modified in their wording; two items were eliminated; and 14 items were incorporated. Finally, a battery of 109 items was obtained (see Annex 1).

Subsequently, we conducted a pilot study with a community sample of 118 participants to ensure an adequate understanding of the items and to identify and select those with good psychometric properties. The battery of items, a sociodemographic questionnaire, and the Informed Consent approved by the Scientific Ethics Committee (CEC) of the Universidad de La Frontera (UFRO) were computerized on the SurveyMonkey platform and disseminated through social networks and email. Of the 109 original items: we eliminated six because they did not meet the statistical criterion for corrected total item correlation greater than 0.3; according to the skewness and kurtosis criteria, we eliminated 26; and for insignificant bivariate correlations, too low or high, we eliminated 40 more. Finally, the CAAS instrument consisted of 32 items in six theoretical dimensions (see Annex 2) with five response options (from 1 = *strongly disagree* to 5 = *strongly agree*). Its psychometric properties will be exposed in the results section.

#### Universal Religious Involment Scale (I-E 12)

This scale, designed to measure religious involvement (Allport and Ross, 1967), was adapted by Carrasco (Carrasco, 2012) for use with Chilean university students. I-e 12 consists of 12 items, 5-point Likert-type (from 1 = *strongly disagree* to 5 = *strongly agree*), and 3 factors: (Festinger, 1964) Intrinsic Orientation (IO); (Galtung, 1990) Extrinsic Social Orientation (OES); and (Kumar et al., 2009) Personal Extrinsic Orientation (PEO). The higher the score, the greater the salience of the religious, social category compared to others, placing religion as a central value in personal identity (OI); higher social gain in terms of interpersonal relationships and status (OES); and greater personal gain, in terms of obtaining protection and consolation (PEO). This structure was adjusted in the Chilean community sample, obtaining a good/excellent internal consistency through McDonald’s Omega coefficient, *Ω _F1_* = 0.916; *Ω _F2_* = 0.964; *Ω _F3_* = 0.872 (Pérez et al., 2022). Likewise, this structure was adjusted in the study sample, considering the correlation between the errors of items 2 and 12 (*X ^2^* = 437.81; *df* = 50; *CFI* = 0.98; *TLI* = 0.97; *RMSEA* = 0.08, 90% CI [0.073, 0.087]); with positive and significant correlations (*p* < 0.001 in all cases) between the factors (F1 and F2: *ρ* = 0.564; F1 and F3: *ρ* = 0.782; F2 and F3: *ρ* = 0.470); and with excellent internal consistency (*Ω _F1_* = 0.949; *Ω _F2_* = 0.974; *Ω _F3_* = 0.924).

### Procedure

We obtained the study sample through the NETQUEST Company under ISO 26362:2009 norm, a data provider for social and market research. Through specialized panels, it offers researchers online study samples that meet the inclusion criteria required by the research. The Informed Consent approved by the Scientific Ethics Committee of La Frontera University was used. The average response time was 21 min.

### Data analysis

First, we use descriptive and frequency statistics to describe the sample. The descriptive analysis of the items (mean, standard deviation, asymmetry, and kurtosis) and the corrected item-total correlation analysis allowed us to determine the discriminative capacity of the items. We consider an indication of threat to said capacity, higher levels of +/− 2 in asymmetry, +/− 7 in kurtosis, and less than 0.3 in corrected item-total correlation (Abad et al., 2011). The Kolmogórov-Smirnov test was used to analyze the normality of the distribution of the scores. We also explore the correlation between elements using Spearman’s Rho correlation to detect extreme levels. Correlations that are too high (greater than 0.8) are an indicator that the items are too similar, and one of them must be removed for redundancy. Correlations that are too low (below 0.3) indicate that one item (or several) does not measure the same construct as the rest, so removing one or more items should be evaluated.

Subsequently, as a statistical strategy to respond to specific objective 2, we carried out a process of cross-validation or replication of the factors in new samples to establish their generalizability. The sample was randomly divided into two sub-samples. First, the relevance of the data for factor analysis was established using the Bartlett index and the Kaiser-Meyer-Olkin (KMO) test in sub-sample 1. Then, we explored the factor structure of the instrument using Exploratory Factor Analysis (EFA), method extraction by unweighted least squares, and oblique rotation. Subsequently, we consider the above criteria for item selection based on corrected item-total correlation, skewness, and kurtosis. In addition, we explore the cross-loadings of the items in the factors, eliminating those with factorial weights greater than 0.3 in two factors (Fabrigar et al., 1999). We also eliminated items with non-significant correlations, below 0.3 or above 0.8, with other items of the same factor.

The resulting structure was replicated and contrasted in sub-sample 2 using Confirmatory Factor Analysis (CFA). It considers the robust unweighted least squares estimator (ULSMV) in a polychoric matrix due to the ordinal nature of the data. In addition, to know more precisely the factorial structure of the instrument, we explore whether this distribution of items by dimensions responds to different models. An oblique model, with two correlated first-order factors (Model 1), or a hierarchical model, with two first-order factors and one second-order factor (Model 2). Also, it was verified if the oblique structure was a better fit than the Exploratory Structural Equations Model (ESEM; Model 3). This structure allows cross-loading between different latent variables or items, since psychological variables have responded better to ESEM than to the assumptions. Restrictive of the CFA(Assis Gomes et al., 2017), or as a bifactor model (Model 4), an alternative to the hierarchical model that considers a general factor that explains the covariation between all the items, at the same time as specific factors or dimensions (Reise, 2012; Rodríguez et al., 2016).

To study the fit of models under analysis (Models 1, 2, 3, and 4), we used the *Root Mean Square Error of Approximation* (*RMSEA*) was considered; and the *Comparative Fit Index* (*CFI*), and the *Tucker-Lewis Index* (*TLI*). A *CFI* and *TLI* ≥ 0.95 and *RMSEA* < 0.05 were considered a good fit; a *CFI* and *TLI* ≥ 0.90 and an *RMSEA* < 0.08 were deemed acceptable. Schwarz’s *Bayesian Information Criterion (BIC)* was also used to compare the models (a lower value indicates a better fit).

To respond to specific objective 3, we used McDonald’s Omega coefficient, a relevant coefficient for use in ordinal scales (Elosua and Zumbo, 2008). Finally, to determine the evidence of the validity of the CAAS based on the relationship with other constructs (specific objective 4), we used Spearman’s correlation coefficient (r_s_) and the Student’s t-test for mean difference with the correction of Welch when group sizes and/or variances are unequal. An intermediate effect size was considered when *d* > 0.05, and large when *d* > 0.08 (Cohen, 1998). We use the statistical packages SPSS 24 for Windows, Mplus 7, Factor 10.9, and JASP.

## Results

### Item analysis

In Annex 2, we collected descriptive data of the items in the study sample. The 32 items showed adequate values of asymmetry or kurtosis. In addition, they revealed a corrected item-total correlation greater than 0.3 with the total scale and the respective theoretical dimension, except for item 1, “*women should not be required to discuss the abortion decision with others*.” For this reason, item 1 was removed from the scale.

In addition, item 32, “*women who choose to abort are brave for challenging the status quo*,” was eliminated. It presents correlations greater than 0.8 with item 17, “*Women who choose abortion are strong for defying the traditional mandate of motherhood*,” and item 25, “*a woman who aborts is a woman with the strength to go against what society expects of her*.” Finally, 30 of the 32 items are maintained in subsequent analyzes (Annex 2).

### Evidence of validity based on the internal structure and ítems analysis of the final scale

With sub-sample 1 (*n* = 611), and considering the 30 items that remain in the instrument, an EFA was performed. The *KMO* index = 0.96, and the Bartlett sphericity test (*χ2*_(435)_ = 10,344, *p* < 0.001) indicate that the correlation matrix is suitable for factor analysis. As a result, we obtained a multidimensional structure of two factors that explain 57.7% of the variance, fulfilling hypothesis two of the study. The first factor of 10 items was called *Autonomy*, the estimable ability of women to act against the voluntary interruption of pregnancy according to their criteria, desire, and life expectatives, regardless of the opinion or desire of others and society. The second factor, of 20 items, was labeled as *Stigma*, discrediting negative attribute manifested through stereotypes, social norms, and discriminatory behaviors directed towards women who attempt to interrupt their pregnancy or have practiced it, marking them internally or externally as inferior to the ideals archetypes of femininity or religious morality.

To obtain a parsimonious instrument, we reviewed the factorial weights and descriptive statistics of the 30 items and selected those with the best qualities for the conformation of the definitive scale. First, we observe that all the items show weights greater than 0.3 in one factor and not in the other. On the other hand, both the *Autonomy factor items* (ranging between 0.443 and 0.732) and the *Stigma factor* (ranging between 0.498 and 0.814) obtained corrected item-total correlation values greater than 0.3. Furthermore, none of the items show extreme asymmetry or kurtosis, so no item is eliminated under these criteria.

*Autonomy* dimension, none presented bivariate correlations higher than 0.8. However, item 3 was eliminated because it showed correlations below 0.3 with two items. The analysis of bivariate correlations for the items of the *Stigma* dimension indicates that item 26 presents high correlations with item 4 and item 10, so it is eliminated from the instrument. Items 2, 8, 20, 30, and 31 are eliminated because they present correlations with other items lower than 0.3. Of the remaining items that maintain correlations between 0.3 and 0.4 with the rest of the items, another four (Hanschmidt et al., 2016; Ministerio de Salud, 2017; Human Rights Committee, 2018; Prusaczyk and Hodson, 2018) are eliminated due to theoretical criteria (all refer to aspects related to erroneous beliefs about health and abortion). As a result, the CAAS is made up of 19 items.

A new analysis of the factorial weights of the items using AFE shows that item 5, “*I believe that girls should have the right to abort*,” presents high weights in both factors (−0.322 in the *Stigma factor* and 0.579 in the *Autonomy factor*). So it is also removed. Finally, the AFE with the resulting list of 18 items (*KMO* = 0.952; Bartlett’s Sphericity test significant, *χ^2^*_(153)_ = 5,964, *p* < 0.001) yields a structure of 2 factors that explain 52.38% of the variance. Table 3 shows the definitive scale, with the descriptive analysis of the items and factorial weights. Annex 3 includes the items of the scale in Spanish. In short, a scale of 18 items is obtained, 8 for *Autonomy* and 10 for *Stigma*. The item with which the participants show a minor agreement is 17, followed by 2 and 4. Item 12 is the one with which they show the greatest deal, followed by 15 and 14.

**Table 3:** Descriptive analysis of definitive items of the CAAS in the sub-sample 1 and factorial weights.

**No.**	**Statement item**	**M**	**SD**	**Skew**	**Kurt**	**CITC-F**	**FW**
**Autonomy**							
05	The woman who decides to abort has self-esteem for giving herself what she wants in life	3.14	1,182	−0.163	−0.626	0.652	0.733
06	A woman has the right to abort as many times as necessary	3.00	1,442	0.011	−1,301	0.635	0.598
09	What a woman wants for her life cannot be truncated by an unwanted pregnancy	3.20	1,311	−0.213	−0.962	0.520	0.572
10	Women who choose abortion are strong for defying the traditional mandate of motherhood	2.91	1,264	0.012	−0.907	0.615	0.813
12	Forcing a woman to carry an unwanted pregnancy to term should be understood as a violation of human rights	3.46	1,358	−0.433	−0.973	0.531	0.417
14	Termination of a pregnancy is justified if necessary for a prime life	3.22	1,263	−0.205	−0.933	0.611	0.690
15	If a woman has no desire to gestate and be a mother, you do not have to do it even if you get pregnant	3.45	1,279	−0.352	−0.911	0.689	0.586
16	A woman who aborts is a woman with the strength to go against what society expects of her	2.87	1,285	0.094	−0.965	0.653	0.806
**Stigma**							
01	I would be disappointed if I knew that someone I love had an abortion	2.23	1,351	0.714	−0.761	0.729	0.769
02	Women should be ashamed to share their decision to abort publicly	1.89	1,146	1,093	0.220	0.674	0.644
03	Many of the women who decide on abortion were not cautious enough to avoid finding themselves in this situation	2.57	1.37	0.330	−1,136	0.657	0.620
04	A woman who aborts is a murderer	1.98	1,229	0.988	−0.135	0.806	0.758
07	Young women take abortion as a game	2.58	1,335	0.25	−1,128	0.732	0.640
08	Women who decide not to abort are blessed by God	2.01	1.20	0.838	−0.365	0.675	0.805
11	Only God can take life	2.40	1,431	0.515	−0.063	0.657	0.622
13	Women from birth have a maternal instinct	2.28	1,226	0.504	−0.822	0.493	0.681
17	Understandably, a man rejects a woman for having had an abortion in the past	1.87	1,051	0.960	0.067	0.598	0.722
18	Women who have abortions do not usually maintain stable relationships	2.21	1,137	0.438	−0.724	0.687	0.719

M = Mean; SD = Standard deviation; Skew = Skewness; Kurt = Kurtosis; CTIC-F = total item correlation corrected by factor; FW = Factor Weights.

**Figure 1:**
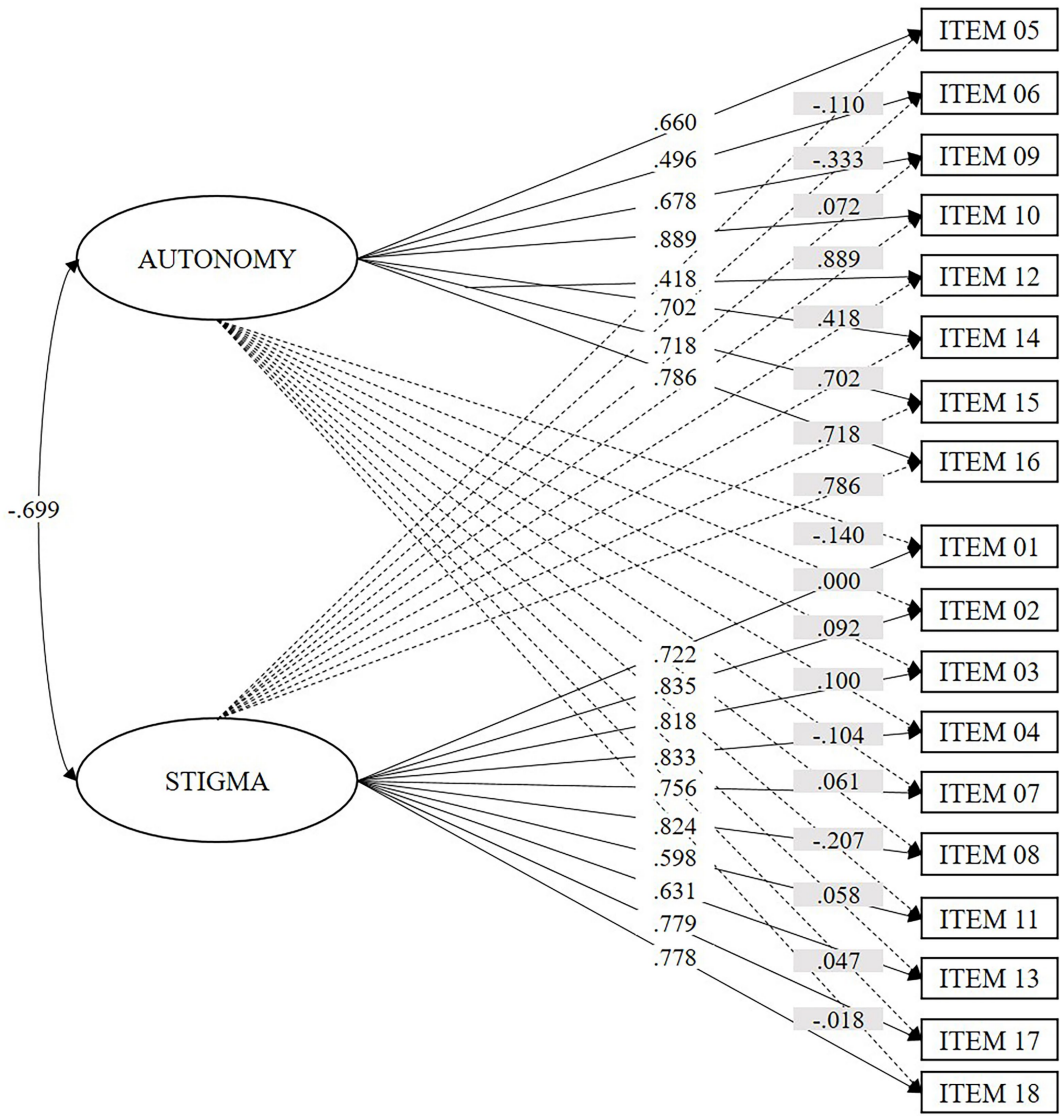
Factorial weights of the items in the ESEM model of two correlated first-order factors (Model 3). Values with a white background represent the factorial weights in the factor; values with a gray background represent the cross-loadings.

Next, we demonstrate the fit of the two-factor model using CFA in sub-sample 2 (*n* = 612). According to the fit indices (see Table 4), the hierarchical model (Model 2) is the one with the worst fit. The oblique model presents an adequate fit (Model 1), but the ESEM and Bifactor models (Models 3 and 4) present better and similar indicators. The correlation between *Autonomy* and *Stigma* (see Figure 1) was inverse and statistically significant (*r* = −0.699; *p* < 0.001).

**Table 4:** Evidence fit of the factor structures in AFC.

**Models**	**χ** ^**2**^	**df**	**CFI**	**RMSEA (90% CI)**	**TLI**	**BIC**
M1. Oblique: 2 correlated first-order factors	568,577	133	0.961	0.073 (0.067–0.079)	0.955	11348.404
M2. Hierarchical: 2 first order factors and a general factor	750,501	136	0.945	0.086 (0.080–0.092)	0.938	11348.404
M3. Oblique: 2 first order factors (ESEM)	263,362	118	0.974	0.063 (0.053–0.074)	0.967	5823.222
M4. Bifactor: 2 first order factors and a general factor	412,956	120	0.974	0.063 (0.057–0.070)	0.967	11348.404

x^2^ = Chi Square; df = degrees of freedom; CFI = Comparative Fit Index; RMSEA = Root Mean Square Error of Approximation; TLI = Tucker-Lewis Index.

### Internal consistency

The McDonald’s Omega coefficient value for the Autonomy factor was 0.908 in sub-sample 1 and 0.892 in sub-sample 2. For the *Stigma factor,* it was 0.941 in sub-sample 1 and 0.937 in sub-sample 2. This is indicative of excellent internal consistency.

### Evidence of validity based on the relationship with other variables

The correlations between the dimensions of the CAAS (*Autonomy* and *Stigma*) and the dimensions of I-E 12 were statistically significant, which confirms hypothesis 4a and demonstrates concurrent validity: *Autonomy*, correlates negatively with Intrinsic Orientation (*ρ* = −0.424; *p* < 0.001), Extrinsic Social Orientation (*ρ* = −0.274; *p* < 0.001) and Personal Extrinsic Orientation (*ρ* = −0.364; *p* < 0.001); and *Stigma* correlates positively with Intrinsic Orientation (*ρ* = 0.571; *p* < 0.001), Personal Extrinsic Orientation (*ρ* = 0.445; *p* < 0.001) and Personal Extrinsic Orientation (*ρ* = 0.448; *p* < 0.001).

We contrasted the Autonomy and Stigma scores to determine discriminant validity using the extreme group comparison strategy. We used the total identification score with different social groups (left political orientation, right political orientation, pro-feminism, pro-LGBTIQ+ rights, pro-euthanasia) to create categorical variables selecting quartile 1 and quartile 4. All groups present statistically significant differences, with effect sizes between intermediate and large, as we stated in hypotheses 4b to 4e (see Table 5).

**Table 5:** Comparison between extreme groups in their scores in the dimensions of the CAAS.

	**Group/Quartile**	**n**	**Min.**	**Max.**	**M**	**SD**	**T**	**df**	**p**	**d**
Autonomy										
	Low left	317	1	5	2,818	1,010	−10.732*	626.06	<0.001	0.84
	High left	327	1	5	3,623	0.888				
	Low right	450	1	5	3,466	0.953	10.665*	933.83	<0.001	0.67
	High right	562	1	5	2,840	0.894				
	Low feminist	537	1	5	2,808	0.840	−11.543*	1,202	<0.001	0.66
	High feminist	686	1	5	3,399	0.947				
	Low LGBTIQ+	610	1	5	2,775	0.859	−14.541*	1219.76	<0.001	0.83
	High LGBTIQ+	613	1	5	3,502	0.891				
	Low euthanasia	499	1	5	2,678	0.825	−15.445*	1,221	<0.001	0.90
	High euthanasia	724	1	5	3,457	0.895				
Stigma										
	Low left	317	1	5	2.44	0.998	10.435*	594.30	<0.001	0.82
	High left	327	1	4.60	1.71	0.771				
	Low right	450	1	4.40	1.74	0.793	−15.591*	1006.05	<0.001	0.97
	High right	562	1	5	2.59	0.931				
	Low feminist	537	1	5	2.56	0.908	12.136*	1132.32	<0.001	0.70
	High feminist	686	1	4.70	1.93	0.877				
	Low LGBTIQ+	610	1	5	2.63	0.888	17.510	1205.45	<0.001	1.00
	High LGBTIQ+	613	1	4.60	1.78	0.797				
	Low euthanasia	499	1	5	2.69	0.888	16.219*	1022.80	<0.001	0.95
	High euthanasia	724	1	4.60	1.87	0.830				

M = Mean; SD = Standard Desviation; df = degrees of freedom; * = Welch’s T test was used.

## Discussion

Community attitudes towards abortion, global and relatively stable evaluations about the VIP and the woman who decides to abort manifested at a cognitive, affective, and/or behavioral level are an indicator of the potential structural, cultural and direct violence that society can exercise against women as a form of gender violence (Galtung, 1990; Kumar et al., 2009; Hessini, 2014). This violence affects women’s mental, physical and reproductive health (American Psychological Association, 2008; Astbury-Ward et al., 2012; McMurtrie et al., 2012; Sorhaindo et al., 2014; Hanschmidt et al., 2016; Ramos, 2016; Mosley et al., 2017; Dides-Castillo and Fernández, 2018; O’Donnell et al., 2018; Moreno López et al., 2019). Therefore, it is essential to have an instrument with good psychometric properties to measure this construct.

The objective of this work was to design a scale to measure attitudes towards abortion and evaluate its psychometric properties in the Chilean community population. As a result, we obtained the CAAS. This scale comprises 18 items with discriminative capacity distributed in 2 factors: *Autonomy* and *Stigma*. CASS had an excellent internal consistency for both factors and maintained the expected relationships with other constructs, demonstrating evidence of concurrent and discriminant validity.

The items that generated the greatest agreement among the participants are part of the *Autonomy dimension*. One of them identifies the prohibition of abortion as a violation of human rights, and the other two refer to respect for the decision of the woman who decides to abort when being a mother is not part of her life plans. The items that generated less agreement belong to the *Stigma* dimension. One of them includes the most severe stereotype among those evaluated, “*a woman who aborts is a murderer*,” and the other two refer to the justification of discriminatory behavior: “*women should be ashamed to share their decision to abort publicly*,” and “*understandably, a man rejects a woman for having had an abortion in the past*.” This result indicates a trend towards greater acceptance of abortion in the study sample, consistent with recent legislative developments in the country: approval levels for free abortion in Chile have been rising recently, from 29% in 2018 to 41% in 2021 (Institut de Publique Sondage d’Opinion Secteur, 2018; Institut de Publique Sondage d’Opinion Secteur, 2020).

As we hypothesized, the CAAS obtained a multidimensional structure. This structure comprises two first-order factors correlated inversely, although it did not coincide with the theoretical structure initially proposed. Items from the theoretical dimensions of plenitude, positive stereotypes, and rights were grouped in the *Autonomy* dimension. In contrast, items from the negative stereotypes, discrimination, and morality dimensions were grouped in the *Stigma* dimension. According to hypothesis three, both factors showed excellent internal consistency. This magnitude is similar to or higher than that reported in most reviewed scales (between 0.60 and 0.96). Among the evaluated models, the ESEM model and the bifactor model showed a better and similar fit. We prefer the ESEM model over the bifactor model because it is the most parsimonious solution and because the factors represent correlated but differentiated constructs from a theoretical perspective. In addition, this model better represents the real behavior of psychological constructs since it allows the indicators to maintain cross-loads with other factors (Assis Gomes et al., 2017). On the other hand, the adequate adjustment of the bifactor model and the level of correlation between the factors (−0.699) could justify using a global score while considering a factor score (Reise, 2012; Rodríguez et al., 2016). However, we do not have theoretical evidence to support the existence of a general factor.

The *Autonomy* construct refers to the level of agreement with the woman’s independence to make decisions about abortion; and the questioning of cultural beliefs about gender that impose motherhood and care over the woman’s will and her plans for life (Bègue, 2001; Kumar et al., 2009; Vitti and Cabello, 2010; Norris et al., 2011; Clements, 2014; Adesse et al., 2016; Prusaczyk and Hodson, 2018). The paternalistic and infantilizing attitudes of the patriarchal system nourish the agreement with the suppression of women’s autonomy (Lagarde, 1994; Osorio, 2022). The woman is seen as a delicate being who needs protection and support, and the woman who decides to have an abortion is seen as unintelligent, inferior, and untrustworthy (Shellenberg et al., 2014; Adesse et al., 2016; Sorhaindo et al., 2016). The previous justifies questioning women’s autonomy to make decisions about their reproductive health (Osborne et al., 2022) in favor of others. For example, community members and health professionals support limiting women’s decision-making capacity in favor of their family and partner (Patel and Johns, 2009; Jozkowski et al., 2018; Alveal-Álamos et al., 2022). Even the woman’s difficulty deciding on her reproductive health is identified as a control tool in abusive relationships (de Las Martin Heras et al., 2015). The *Autonomy* dimension is represented in other scales, such as the *Abortion Attitudes Scale* (Stets and Leik, 1993), the *Abortion as a Right Scale* (Rominski et al., 2017), the *CLASS* (Sorhaindo et al., 2016), *and the CAAI* (Marván et al., 2018), which accounts for the concern for this construct in other cultural realities.

*Stigma* dimension collects the community’s agreement with stereotypes and social norms about femininity and morality that mark women who abort as inferior and justify discriminatory treatment (McMurtrie et al., 2012; Shellenberg et al., 2014; Sorhaindo et al., 2014; Adesse et al., 2016; Hanschmidt et al., 2016; Sorhaindo et al., 2016). This factor is represented in scales such as the *Abortion attitudes scale* (Stets and Leik, 1993), the *SABAS* (Shellenberg et al., 2014), the *CLASS* (Sorhaindo et al., 2016), or the *AAAPPS* (Martin et al., 2020). Thus, are measured in this factor: stereotyped ideas such as the woman who aborts are not very cautious, a murderer, promiscuous, and libertine (Shellenberg et al., 2014; Adesse et al., 2016; Sorhaindo et al., 2016; Pérez et al., 2020); discriminatory beliefs such as that abortion is a shameful action that should be carried out in secret (McMurtrie et al., 2012; Hanschmidt et al., 2016); and conservative ideas, such as that the woman who decides not to have an abortion is morally superior in the eyes of God, that life must be respected from conception (Piazza, 2012; Pfeffer, 2017; Sorhaindo et al., 2016), or that motherhood is an instinct (Lagarde, 1994; Kumar et al., 2009; Osorio, 2022). This factor may be especially relevant to work on preventing direct violence against women since, as we said before, groups and individuals under these stereotypes and beliefs exercise violence through threats, deception, discriminatory treatment, and disqualification (Morgan, 2017; Jardim and Modena, 2018; Williams et al., 2018; Lowe, 2019; Lowe and Page, 2019; Makleff et al., 2019; Pérez-Arredondo and Graells-Garrido, 2021).

The literature identifies religion and conservative political orientation as the most relevant correlates of attitudes towards abortion (Patev et al., 2019a,b; Pérez et al., 2020; Cutler et al., 2021; Osborne et al., 2022; Pérez et al., 2022). As hypothesized, our results support this premise since a higher score in Intrinsic Orientation (IO), Extrinsic Social Orientation (ESO), and Personal Extrinsic Orientation (PEO) correlates negatively with *Autonomy* and positively with *Stigma* (H4a), with the strongest correlation being with OI in both cases. These results imply that the participants that obtain social and personal gain from identifying themselves as religious (ESO and PEO), and above all, for whom religious identity prevails over other social identities to regulate and guide their behavior (IO; Allport and Ross, 1967), accept women’s autonomy to decide on abortion to a lesser extent and are more in agreement with stereotypes and stigmatizing beliefs about women who abort, and with discriminatory behavior towards them. This result is evidence of concurrent validity. In addition, the groups of participants with low levels of identification with a left-wing political orientation (H4b), and high levels of identification with a right-wing political orientation (H4c), obtain a lower mean score in *Autonomy* and a higher mean score in *Stigma*. These are evidence of the discriminant validity of the scale. These results make sense that both groups take the same values and beliefs about gender and the beginning of the life we have reviewed, as a guide to define morally acceptable behavior (Kumar et al., 2009; Piazza, 2012; Clements, 2014; Sorhaindo et al., 2014; Hanschmidt et al., 2016; Pfeffer, 2017; Pérez et al., 2020, 2022). Consequently, religious and politically conservative people question women’s autonomy and evaluate them as inferior to the ideals of femininity and morality when they transgress these social norms, these groups being the historical promoters of laws that limit access to abortion, also in Chile (Dides-Castillo and Fernández, 2018; Elgueta et al., 2019; Maira et al., 2019; Osorio, 2022).

Finally, and as further evidence of the discriminant validity of the scale, the groups of participants with low levels of identification with the average feminist person (H4d), LGBTIQ+ rights defender (H4e), and euthanasia (H4f), obtain lower average scores on *Autonomy*, and higher in *Stigma*. This result is empirical evidence that supports the idea formulated by the Bellagio group: whoever questions the right to abortion also questions other doctrines, rights, or individual freedoms (Hessini, 2014). What has been said is consistent with the rest of the results to the extent that, like the VIP, feminism and the LGBTIQ+ community threaten the traditional gender order that establishes socially accepted behavior for women and non-binary people (Lagarde, 1994; Janssen and Scheepers, 2019; Hernandez, 2021). At the same time, the acceptance of euthanasia, in the same way as abortion, means the violation of the norm of religious morality on respect for life from conception to natural death (Stets and Leik, 1993; Pfeffer, 2017; Francis et al., 2019).

We must consider some aspects, like limitations of the study, that may affect the scope of the results. In the first place, although the study sample is balanced according to gender, age, and socioeconomic level, this balance is not representative of the population distribution in Chile (Instituto Nacional de Estadísticas, 2018). In addition, the sample has been collected through an online panel, which translates into a bias: there are mostly participants with Internet access and good command of new technologies. On the other hand, the CAAS is a measure of self-reported explicit attitudes, which may be affected by social desirability, considering that abortion is a controversial issue. Finally, it should be noted that this study offers psychometric evidence for its use in the Chilean population, but it is necessary to accumulate more evidence to guarantee its use, such as, for example, its predictive validity on support for abortion access policies or direct violent behavior. In addition, the evidence of validity accumulated in this study on the relationship of the scale with other variables is based on single-item measurements. Exploring other validity evidence in future research and its applicability in specific populations, such as health professionals, is recommended. Due to their direct dealings with women who request VIP, health professionals are in a privileged position to exercise violence (Jardim and Modena, 2018; Williams et al., 2018; Makleff et al., 2019). In addition, as another future line of research, we propose to explore implicit measures and within-subject designs to assess attitudes toward abortion since they have been shown to reflect a greater extent the personal attitudes of the individual (Sakaluk and Milhausen, 2012) than the explicit attitudes.

In conclusion, this work provides the first scale that evaluates attitudes towards abortion in Chile. The CAAS is an adequate tool for use with the Chilean community population, with evidence of validity in its internal structure, concurrent and discriminant validity, and excellent internal consistency. Our results indicate that this scale presents two correlated but differentiated factors, *Autonomy,* and *Stigma*, with religious participants and those with a conservative political orientation who question women’s autonomy to a greater extent and are more in agreement with the stigmatization of abortion. In addition, those who have a restrictive view of abortion do not identify as pro-feminists, pro-LGBTIQ+, or pro-euthanasia. Based on the results, we recommend using this instrument to understand the population’s attitudes in the country, identify individuals with greater potential to exercise direct violence, and contribute to developing intervention and prevention programs.

## Data availability statement

The raw data supporting the conclusions of this article will be made available by the authors, for readers who request it.

## Ethics statement

The studies involving human participants were reviewed and approved by Comité Ético Científico de la Universidad de La Frontera. The patients/participants provided their written informed consent to participate in this study.

## Author contributions

BP, JJB and FR: conceptualization. BP and AC-S: methodology. BP, JJB, CA-A, and LJ: fieldwork. BP, JJB, and AC-S: formal analysis. BP and JJB: writing—original draft preparation. BP, AC-S, CA-A, and FR: writing—review and editing. BP: project administration. FR: Formulation of research proposal and initial manuscript, international development of the line and revision of the manuscript. All authors contributed to the article and approved the submitted version.

## Funding

This study is part of the project FONDECYT/No.11180588. This work was funded by the European Regional Development Funds (European Union and Principality of Asturias) through the Science, Technology, and Innovation Plan (AYUD/2021/51411).

## Conflict of interest

The authors declare that the research was conducted in the absence of any commercial or financial relationships that could be constructed as a potential conflict of interest.

## Publisher’s note

All claims expressed in this article are solely those of the authors and do not necessarily represent those of their affiliated organizations, or those of the publisher, the editors and the reviewers. Any product that may be evaluated in this article, or claim that may be made by its manufacturer, is not guaranteed or endorsed by the publisher.

## Supplementary material

The Supplementary material for this article can be found online at: https://www.frontiersin.org/articles/10.3389/fpsyg.2022. 1008492 z/full#supplementary-material

## References

[ref1] Abad F. J. Olea J. Ponsoda V. García C. (2011). Medición en ciencias sociales y de la Salud. Síntesis.

[ref2] Adesse L. Jannotti C. B. da Silva K. S. Fonseca V. M. (2016). Aborto e estigma: uma análise da produção científica sobre a temática. Ciênc Saúde Coletiva. 21, 3819–3832. doi: 10.1590/1413-812320152112.07282015, PMID: 27925122

[ref3] Allport G. W. Ross J. M. (1967). Personal religious orientation and prejudice. J. Pers. Soc. Psychol. 5, 432–443. doi: 10.1037/h0021212, PMID: 6051769

[ref4] Alveal-Álamos C. Pérez B. Obando A. Carte L. Jara L. (2022). La Objeción de Conciencia frente a la Interrupción Voluntaria del Embarazo: Motivaciones que traspasan las Creencias Morales y Religiosas en Profesionales de la Salud Chilenos. Rev Punto Género. 17, 307–344.

[ref5] American Psychological Association (2008). “Task force on mental health and abortion,” in Report of the task force on mental health and abortion [internet] (Washington, DC).

[ref6] Assis Gomes C. M. Almeida L. S. Núñez J. C. (2017). Rationale and applicability of exploratory structural equation modeling (ESEM) in psychoeducational contexts. Psicothema 29.3, 396–401. doi: 10.7334/psicothema2016.369 28693713

[ref7] Astbury-Ward E. Parry O. Carnwell R. (2012). Stigma, abortion, and disclosure—findings from a qualitative study. J. Sex. Med. 9, 3137–3147. doi: 10.1111/j.1743-6109.2011.02604.x 22239919

[ref8] Ato M. López-García J. J. Benavente A. (2013). Un sistema de clasificación de los diseños de investigación en psicología. An Psicol. 29, 1038–1059. doi: 10.6018/analesps.29.3.178511

[ref9] Bahr S. J. Marcos A. C. (2003). Cross-cultural attitudes toward abortion: Greeks versus Americans. J. Fam. Issues 24, 402–424. doi: 10.1177/0192513X02250892, PMID: 15871159

[ref10] Bègue L. (2001). Social judgment of abortion: a black-sheep effect in a Catholic sheepfold. J. Soc. Psychol. 141, 640–649. doi: 10.1080/00224540109600577, PMID: 11758041

[ref11] Carrasco C. (2012). Orientación religiosa y sintomatología depresiva en estudiantes de la Universidad del Bío-Bío [Internet]. [Región del Bío-Bío]: Universidad del Bío-Bío. Available from: https://fdocuments.es/document/orientacin-religiosa-y-sintomatologa-depresiva-en-de-acuerdo-con-la-teora.html?page=1

[ref12] Clements B. (2014). Religion and the sources of public opposition to abortion in Britain: the role of ‘belonging’, ‘behaving’ and ‘believing’. Sociology 48, 369–386. doi: 10.1177/0038038513490354

[ref600] Cohen J. (1988). Statistical power analysis for the behavioral sciences. 2nd Edn. Lawrence Erlbaum Associates, Publishers.

[ref13] Cutler A. S. Lundsberg L. S. White M. A. Stanwood N. L. Gariepy A. M. (2021). Characterizing community-level abortion stigma in the United States. Contraception 104, 305–313. doi: 10.1016/j.contraception.2021.03.021, PMID: 33789081

[ref14] de Las Martin Heras S. Velasco C. de Luna J. D. Martin A. (2015). Unintended pregnancy and intimate partner violence around pregnancy in a population-based study. Women. Birth 28, 101–105. doi: 10.1016/j.wombi.2015.01.003, PMID: 25622887

[ref16] Dides-Castillo C. Fernández C. (2018). DOSSIER SOBRE EL ABORTO EN LATINOAMÉRICA. Aborto en Chile: avances en derechos humanos 43, 61–76.

[ref17] Donoso E. Vera C. (2016). El aborto en Chile: aspectos epidemiológicos, históricos y legales. Rev. Chil. Obstet. Ginecol. 81, 534–545. doi: 10.4067/S0717-75262016000600014

[ref18] Elgueta R. Santoni A. Fediakova E. (2019). La persistencia de la fe: cambios y vigencia del clivaje político-religioso en Chile (1938-2017). Estud Ibero-Am. 45, 149–162. doi: 10.15448/1980-864X.2019.2.31335

[ref19] Elosua P. Zumbo B. C. (2008). Coeficientes de fiabilidad para escalas de respuesta categórica ordenada. Psicothema 20, 896–901. 18940100

[ref20] Fabrigar L. R. Wegener D. T. MacCallum R. C. Strahan E. J. (1999). Evaluating the use of exploratory factor analysis in psychological research. Psychol. Methods 4, 272–299.

[ref21] Festinger L. (1964). Conflict, decision and dissonance. U. Press. Stanford.

[ref22] Francis L. J. McKenna U. Sahin A. (2019). “Religion, human rights and matters of life and death: exploring attitude toward abortion and euthanasia among adolescents in England and Waleso,” in Euthanasia, abortion, death penalty and religion - the right to life and its limitations religion and human rights. eds. Ziebertz H. G. Zaccaria F. (Cham: Springer).

[ref23] Frez J. (2018). Implementación de la Ley 21.030 en el Hospital de Puerto Montt. Cuad Méd Soc. 58, 83–85.

[ref24] Galtung J. (1990). Cultural Violence. J Peace Rsearch. 27, 291–305. doi: 10.1177/0022343390027003005

[ref25] García C. (2012). Actitudes hacia el aborto legal asistido. Doc Trab Soc. 50, 269–279.

[ref26] Hanschmidt F. Linde K. Hilbert A. Riedel-Heller S. G. Kersting A. (2016). Abortion stigma: a systematic review: abortion stigma - a systematic review. Perspect. Sex. Reprod. Health 48, 169–177. doi: 10.1363/48e8516, PMID: 27037848

[ref27] Hendriks J. (2012). Scale construction utilising the Rasch unidimensional measurement model: a measurement of adolescent attitudes towards abortion. Australas Med J 5, 251–261. doi: 10.4066/AMJ.2012.952, PMID: 22848320 PMC3395283

[ref28] Hernandez A. D. (2021). Intersections of feminist identification and hostile sexism. J. Sci. Study Relig. 60, 27–45. doi: 10.1111/jssr.12694

[ref29] Hessini L. (2014). A learning agenda for abortion stigma: recommendations from the Bellagio expert group meeting. Women Health 54, 617–621. doi: 10.1080/03630242.2014.919987, PMID: 25062399

[ref30] Hill A. (2004). The relationship between attitudes about abortion and cognitive complexity UW-J Undergrad Res VII, 1–6.

[ref31] Holcombe S. J. Burrowes S. Hailu D. Scott R. Berhe A. (2018). Professional pragmatism and abortion stigma: assessing the performance of the stigmatizing attitudes, beliefs and actions scale (SABAS) among Ethiopian midwives. Afr. J. Reprod. Health 22, 26–39. doi: 10.29063/ajrh2018/v22i2.3, PMID: 30052331

[ref32] Human Rights Committee. (2018). General comment no. 36 (2018) on article 6 of the international covenant on civil and political rights, on the right to life [internet]. Available from: https://www.safeabortionwomensright.org/news/un-human-rights-committee-general-comment-no-36-2018-on-article-6-of-the-international-covenant-on-civil-and-political-rights-on-the-right-to-life/

[ref33] Institut de Publique Sondage d’Opinion Secteur. (2018). Informe sobre la aceptación del aborto [Internet]. Available from: https://www.ipsos.com/sites/default/files/ct/news/documents/2018-04/ipsos_public_affairs_aborto_2018.pdf

[ref34] Institut de Publique Sondage d’Opinion Secteur. (2020). Miradas globales sobre el aborto. Favorabilidad hacia la legalización del aborto [Internet]. Available from: https://www.ipsos.com/es-cl/68-de-los-chilenos-estan-favor-del-aborto

[ref35] Instituto Nacional de Estadísticas. (2018). Síntesis de Resultados Censo 2017 [Internet]. Available from: https://shortest.link/3L1t

[ref36] Janssen D. J. Scheepers P. (2019). How religiosity shapes rejection of homosexuality across the globe. J. Homosex. 66, 1974–2001. doi: 10.1080/00918369.2018.1522809, PMID: 30372378

[ref37] Jardim D. M. B. Modena C. M. (2018). Obstetric violence in the daily routine of care and its characteristics. Rev Lat Am Enfermagem [Internet]. [cited 2022 Jul 26]; 26. Available from: http://www.scielo.br/scielo.php?script=sci_arttext&pid=S0104-11692018000100613&lng=en&tlng=en 10.1590/1518-8345.2450.3069 PMC6280177 30517571

[ref38] Jozkowski K. N. Crawford B. L. Hunt M. E. (2018). Complexity in attitudes toward abortion access: results from two studies. Sex. Res. Soc. Policy 15, 464–482. doi: 10.1007/s13178-018-0322-4

[ref39] Kumar A. Hessini L. Mitchell E. M. H. (2009). Conceptualising abortion stigma. Cult. Health Sex. 11, 625–639. doi: 10.1080/13691050902842741, PMID: 19437175

[ref40] Lagarde M. (1994). Perspectiva de género. Diakonia. 71, 23–29.

[ref41] Lizotte M. K. (2015). The abortion attitudes paradox: model specification and gender differences. J Women Polit Policy. 36, 22–42. doi: 10.1080/1554477X.2015.985151

[ref42] Lowe P. (2019). (re)imagining the ‘backstreet’: anti-abortion campaigning against decriminalisation in the UK. Sociol. Res. Online 24, 203–218. doi: 10.1177/1360780418811973

[ref43] Lowe P. Page S. J. (2019). Rights-based claims made by UK anti-abortion activists. Health Hum Rights J. 21, 133–144. PMC6927388 31885443

[ref44] Maira G. Casas L. Vivaldi L. (2019). Abortion in Chile: the long road to legalization and its slow implementation. Health Hum. Rights 21, 121–131. 31885442 PMC6927382

[ref45] Makleff S. Labandera A. Chiribao F. Friedman J. Cardenas R. Sa E.. (2019). Experience obtaining legal abortion in Uruguay: knowledge, attitudes, and stigma among abortion clients. BMC Womens Health 19:155. doi: 10.1186/s12905-019-0855-6, PMID: 31815617 PMC6902415

[ref46] Marshall P. Zúñiga Y. (2020). Objeción de conciencia y aborto en Chile. Rev Fac Derecho. 84, 99–130.

[ref47] Martin L. A. Seewald M. Johnson T. R. B. Harris L. H. (2020). Trusted colleagues or incompetent hacks? Development of the attitudes about abortion-providing physicians scale. Womens Health Issues 30, 16–24. doi: 10.1016/j.whi.2019.09.002 31668561

[ref48] Marván M. L. Lagunes-Córdoba R. Orihuela-Cortés F. (2018). Diseño de un cuestionario de actitudes hacia el aborto inducido. Salud Pública México. 60:742. doi: 10.21149/9182 30699283

[ref49] McMurtrie S. M. García S. G. Wilson K. S. Diaz-Olavarrieta C. Fawcett G. M. (2012). Public opinion about abortion-related stigma among Mexican Catholics and implications for unsafe abortion. Int. J. Gynecol. Obstet. 118, S160–S166. doi: 10.1016/S0020-7292(12)60016-2, PMID: 22920621

[ref50] Ministerio de Salud. (2017). Ley N° 21.030 de 2017. Regula la despenalización de la interrupción voluntaria del embarazo en tres causales [Internet]. Available from: https://www.bcn.cl/leychile/navegar?idNorma=1108237

[ref51] Moreno López M. Flores Celis K. González-Forteza C. Saltijeral M. T. Schiavon R. Collado M. E.. (2019). Relationship between perceived stigma and depressive symptomatology in women who legally interrupt pregnancy in Mexico City. Salud Ment. 42, 25–32. doi: 10.17711/SM.0185-3325.2019.004

[ref52] Morgan L. (2017). The Dublin declaration on maternal health care and anti-abortion activism: examples from Latin America. Health Hum Rights J. 19, 41–53. PMC5473037 28630540

[ref53] Mosley E. A. Anderson B. A. Harris L. H. Fleming P. J. Schulz A. J. (2020). Attitudes toward abortion, social welfare programs, and gender roles in the U. S. and South Africa. Crit. Public Health 30, 441–456. doi: 10.1080/09581596.2019.1601683 PMC8975127 35368244

[ref54] Mosley E. A. King E. J. Schulz A. J. Harris L. H. De Wet N. Anderson B. A. (2017). Abortion attitudes among south Africans: findings from the 2013 social attitudes survey. Cult. Health Sex. 19, 918–933. doi: 10.1080/13691058.2016.1272715, PMID: 28100112 PMC5849464

[ref55] Muñoz P. Parrini J. Dresdner R. Jiménez M. (2021). Dilemas clínicos en la constitución de la tercera causal de la interrupción voluntaria del embarazo. Rev Médica Chile. 149, 758–764. doi: 10.4067/s0034-98872021000500758, PMID: 34751329

[ref56] Nicholls L. Cuestas F. (2018). Penalización del aborto: violencia política y abusos de la memoria en Chile. Artigo. 27, 367–380. doi: 10.1590/s0104-12902018170419

[ref57] Norris A. Bessett D. Steinberg J. R. Kavanaugh M. L. De Zordo S. Becker D. (2011). Abortion stigma: a reconceptualization of constituents, causes, and consequences. Womens Health Issues 21, S49–S54. doi: 10.1016/j.whi.2011.02.010, PMID: 21530840

[ref58] O’Donnell A. T. O’Carroll T. Toole N. (2018). Internalized stigma and stigma-related isolation predict Women’s psychological distress and physical health symptoms post-abortion. Psychol. Women Q. 42, 220–234. doi: 10.1177/0361684317748937

[ref59] Osborne D. Huang Y. Overall N. C. Sutton R. M. Petterson A. Douglas K. M.. (2022). Abortion attitudes: an overview of demographic and ideological differences. Polit. Psychol.:pops.12803. doi: 10.1111/pops.12803

[ref60] Osorio C. M. (2022). Las mujeres y la crisis sobre su autonomía. Reflexiones en torno a los cuerpos que abortan y la nueva institucionalidad política. Bol Onteaiken. 33, 70–80.

[ref61] Patel C. J. Johns L. (2009). Gender role attitudes and attitudes to abortion: are there gender differences? Soc. Sci. J. 46, 493–505. doi: 10.1016/j.soscij.2009.02.006

[ref62] Patev A. J. Hall C. J. Dunn C. E. Bell A. D. Owens B. D. Hood K. B. (2019b). Hostile sexism and right-wing authoritarianism as mediators of the relationship between sexual disgust and abortion stigmatizing attitudes. Personal. Individ. Differ. 151:109528. doi: 10.1016/j.paid.2019.109528

[ref63] Patev A. J. Hood K. B. Hall C. J. (2019a). The interacting roles of abortion stigma and gender on attitudes toward abortion legality. Personal. Individ. Differ. 146, 87–92. doi: 10.1016/j.paid.2019.04.005

[ref64] Pérez B. Concha-Salgado A. Aburto-González V. Mandiola-Sandoval C. Muñoz-Henríquez C. Cerda-Muñoz D. (2022). Religiosity, abortion stigma and the mediating effect of gender attitudes. A study in the Chilean population (*Religiosidad, estigma del aborto, y el efecto mediador de las actitudes de género. Un estudio en población chilena*). Int J Soc Psychol. 37, 211–241. doi: 10.1080/02134748.2022.2034290

[ref65] Pérez B. Sagner-Tapia J. Elgueta H. E. (2020). Despenalización del aborto en Chile: una aproximación mixta desde la percepción del aborto en población comunitaria. Gac. Sanit. 34, 485–492. doi: 10.1016/j.gaceta.2018.11.004, PMID: 30583975

[ref66] Pérez-Arredondo C. Graells-Garrido E. (2021). Twitter and abortion: online hate against pro-choice female politicians in Chile. J Lang Aggress Confl. 9, 127–154. doi: 10.1075/jlac.00056.per

[ref67] Pfeffer B. (2017). Abortion, moral Law, and the first amendment: The conflict between Fetal Rights & Freedom of religion. William Mary J. Women Law. 23:271–335.

[ref68] Piazza J. (2012). “If you love me keep my commandments”: religiosity increases preference for rule-based moral arguments. Int. J. Psychol. Relig. 22, 285–302. doi: 10.1080/10508619.2011.638598

[ref69] Prusaczyk E. Hodson G. (2018). Left-right differences in abortion policy support in America: clarifying the role of sex and sexism in a nationally representative 2016 sample. Personal. Individ. Differ. 127, 22–25. doi: 10.1016/j.paid.2018.01.030

[ref70] Ramos S. (2016). Investigación sobre aborto en América Latina y el Caribe. Una agenda renovada para informar políticas públicas e incidencia (resumen ejecutivo). Estud Demográficos Urbanos. 31:29.

[ref71] Reise S. P. (2012). The rediscovery of Bifactor measurement models. Multivar. Behav. Res. 47, 667–696. doi: 10.1080/00273171.2012.715555, PMID: 24049214 PMC3773879

[ref72] Robledo P. (2018). Desafíos pendientes en la implementación de la Ley 21.030 de Chile, que despenalizó la interrupción voluntaria del embarazo. Cuad Méd Soc. 58, 73–82.

[ref73] Rodríguez A. Reise S. P. Haviland M. G. (2016). Evaluating bifactor models: calculating and interpreting statistical indices. Psychol. Methods 21, 137–150. doi: 10.1037/met0000045, PMID: 26523435

[ref74] Rominski S. D. Darteh E. Dickson K. S. Munro-Kramer M. (2017). Attitudes toward abortion among students at the University of Cape Coast. Ghana. Sex Reprod Healthc. 11, 53–59. doi: 10.1016/j.srhc.2016.10.002, PMID: 28159129

[ref75] Sahar G. Karasawa K. (2005). Is the personal always political? A cross-cultural analysis of abortion attitudes. Basic Appl. Soc. Psychol. 27, 285–296. doi: 10.1207/s15324834basp2704_1

[ref76] Sakaluk J. Milhausen R. (2012). Factors influencing university students’ explicit and implicit sexual double standards. J. Sex Res. 49, 464–476. doi: 10.1080/00224499.2011.569976, PMID: 21534028

[ref77] Shellenberg K. M. Hessini L. Levandowski B. A. (2014). Developing a scale to measure stigmatizing attitudes and beliefs about Women who have abortions: results from Ghana and Zambia. Women Health 54, 599–616. doi: 10.1080/03630242.2014.919982, PMID: 25074064

[ref78] Snegroff S. (1976). The development of instruments to measure attitudes toward abortion and knowledge of abortion. J. Sch. Health 46, 273–277. doi: 10.1111/j.1746-1561.1976.tb02015.x, PMID: 1046505

[ref79] Sorhaindo A. M. Juárez-Ramírez C. Olavarrieta C. D. Aldaz E. Mejía Piñeros M. C. Garcia S. (2014). Qualitative evidence on abortion stigma from Mexico City and five states in Mexico. Women Health 54, 622–640. doi: 10.1080/03630242.2014.919983, PMID: 25068848

[ref80] Sorhaindo A. M. Karver T. S. Karver J. G. Garcia S. G. (2016). Constructing a validated scale to measure community-level abortion stigma in Mexico. Contraception 93, 421–431. doi: 10.1016/j.contraception.2016.01.013, PMID: 26825257

[ref81] Stets J. E. Leik R. K. (1993). Attitudes about abortion and varying attitude structures. Soc. Sci. Res. 22, 265–282. doi: 10.1006/ssre.1993.1013

[ref82] Vitti D. Cabello M. (2010). A religião e o discurso de mulheres sobre o abortamento. Psicol Teor E Pesqui. 26, 193–196. doi: 10.1590/S0102-37722010000100021

[ref83] Williams C. Jerez C. Klein K. Correa M. Belizán J. Cormick G. (2018). Obstetric violence: a Latin American legal response to mistreatment during childbirth. BJOG Int. J. Obstet. Gynaecol. 125, 1208–1211. doi: 10.1111/1471-0528.15270, PMID: 29727059

