# Supplementary material for: Design and study of psychometric properties of the Community Attitude to Abortion Scale (CAAS) with the Chilean population: Autonomy and Stigma
Source: Front Psychol. 2022 Dec 23;13:1008492. doi: 10.3389/fpsyg.2022.1008492 (PMC9822711; doi:10.3389/fpsyg.2022.1008492)
Supplement: Supplementary file 1 [file Data_Sheet_1.PDF]

Supplementary Material

1 Supplementary Figures and Tables

Annex 1

Descriptive statistics and corrected total item correlation (with complete scale and by theoric dimensions) of the CAAS in a pilot sample (N = 118)

Items		M	SD	Skew	Kurt	CTIC CS	CITC TD
The prime of your life							
3	Women who decide to abort take care of their mental health	2.83	1.10	0.304	-.188	.598	.628
22	Understandably, a woman decides to have an abortion because she is concerned about her present and/or future personal development.	1.99	1.30	1,186	.206	.787	.716
57*	What a woman wants for her life cannot be truncated by an unwanted pregnancy	2.20	1.26	.820	-.370	.595	.629
69	It's okay for a woman to have an abortion just because she doesn't want to have a child right now	2.09	1.41	1,028	-.383	.769	.819
73*	Termination of a pregnancy is justified if necessary for a prime life	2.28	1.41	.838	-.627	.625	.678
90*	If a woman has no desire to gestate and be a mother, you don't have to do it even if you get pregnant	1.83	1.27	1,421	.793	.837	.803
101	It is okay for a woman to decide to have an abortion to protect the well-being of her family and her current children	2.19	1.37	.851	-.506	.738	.817
107	Who decides to abort is generous with herself and her life plans	2.62	1.33	.533	-.756	.657	.735
Positive Stereotypes							
1	A woman who aborts knows what her rights are	2.86	1.13	-.089	-.668	.298	.323
4	Women who abort because they feel they are not ready to raise a child are responsible women	2.03	1.29	1,188	.3. 4. 5	.817	.745
14*	The decision to terminate a pregnancy is a sign of parental responsibility	2.31	1.38	.839	-.51	.714	.737
21	Women usually inform themselves responsibly about the healthiest way to terminate their pregnancy	2.95	1.13	-.007	-.619	.325	.378
37*	The woman who decides to abort has self-love for giving herself what she wants in life	2.57	1.19	.372	-.675	.660	.742
47	A woman who chooses to terminate her pregnancy is a determined person who knows what she wants for her life	2.63	1.13	.273	-.51	.607	.718
62*	Women who choose abortion are strong for defying the traditional mandate of motherhood	2.97	1.38	.082	-1,107	.600	.699
91*	A woman who aborts is a woman with the strength to go against what society expects of her	2.75	1.30	.327	-.82	.566	.783
109 *	Women who choose to abort are brave for challenging the status quo	2.83	1.35	0.251	-.988	.594	.735
Entitlement							
6	The woman should be able to abort if she decides to, whatever the reason	1.74	1.31	1,567	.949	.867	.866
9*	Women should not be required to discuss the abortion decision with others	2.14	1.38	0.975	-.383	.622	.643
20*	I believe that girls should have the right to abort	1.86	1.30	1,423	.810	.766	.773
30	Abortion should be prohibited in any circumstance	1.42	1.01	2.56	5.68	.749	.724
32	In abortion, the only one who should decide is the woman.	2.21	1.33	.810	-.507	.79	.835
35	A Law of abortion by causes is the best alternative	2.25	1.16	.647	-.501	.302	.364
38*	A woman has the right to abort as many times as necessary	2.35	1.41	.736	-.763	.779	.788
42	Without a law that sets limits and penalties, abortion would become a whimsical practice for women	1.66	1.18	1,678	1.62	.834	.791
48	The criminalization of abortion violates the reproductive rights of women	1.94	1.33	1,267	.303	.773	.778

51	Women who decide to abort should need the approval of doctors	2.05	1.29	.941	-.411	.586	.588
60	Defending the right to life of the unborn is what the State should do	1.97	1.33	1,112	-.05	.831	.837
65*	Forcing a woman to carry an unwanted pregnancy to term should be understood as a violation of human rights	1.95	1.33	1,263	.275	.736	.724
66	To perform an abortion, the health professional should have the approval of both parents	2.09	1.29	.931	-.294	.796	.847
70	Women should have their children no matter how they were conceived	1.58	1.11	1.85	2,201	.773	.734
78	From the moment of conception, the zygote is a human being with rights	1.98	1.36	1,154	.022	.843	.862
82	The couple should have the same weight in the decision to perform an abortion	2.19	1.36	.758	-.745	.659	.738
83	Girls and young women who consider abortion should need the approval of their parents	2.05	1.30	1,044	-.062	.734	.789
84	Women should have access to abortion in public hospitals	1.58	1.10	1971	2,966	.838	.802
85	Abortion is a matter of two: both the man and the woman must decide	2.21	1.33	.743	-.638	.688	.749
87	Women have the right to decide about their own body	1.54	1.08	2,228	4,166	.773	.719
104	Abortion should be part of human rights	1.94	1.34	1,233	.187	.87	.855
Negative Stereotypes							
2	Women who decide to abort don't know what they're up against	2.38	1.20	.589	-.497	.498	.486
5	Arguing having suffered a rape to request an abortion is a frequent excuse	2.73	1.26	.188	-.832	.218	.228
10*	Women who do not hesitate to abort are radical feminists	1.58	.94	2,023	4,312	.583	.598
23	Young women abort more	2.86	.90	.272	.304	-.180	-.249
31*	A woman carries the trauma of abortion all her life	2.92	1.20	.045	-.609	.568	.563
33*	Many of the women who decide on abortion were not cautious enough to avoid finding themselves in this situation	2.07	1.22	.88	-.378	.714	.687
34*	A woman who aborts is a murderer	1.52	1.10	2,075	3,135	.827	.712
36	There are quite a few women who use abortion as a contraceptive method	1.72	1.01	1,193	.553	.418	.440
44	A woman who has an abortion is a bad mother	1.34	.85	2,858	8,200	.768	.699
45	Women who decide to abort are crazy	1.23	.65	3,421	13,159	.794	.744
50*	Young women take abortion as a game	1.64	1.11	1,827	2,529	.734	.733
59	A woman who has had an abortion may encourage other women to abort	2.11	1.12	.568	-.635	.402	.451
67*	A girl should consider other alternatives to abortion to avoid trauma	2.19	1.32	.717	-.777	.745	.678
68	Women who abort once, will probably do it again	2.18	1.05	.489	-.412	.514	.553
75*	The health of a woman who has an abortion is never as good as it was before the abortion	1.97	1.13	.850	-.176	.653	.688
77	Low-income women have more abortions	2.15	1.08	.471	-.602	.236	.361
92	Any woman can find herself in a situation where she has to decide whether to have an abortion or not.	1.68	1.05	1,752	2,561	.619	.544
93	Women who decide to abort are cowards for taking the easy way out	1.47	.960	2,238	4,701	.811	.786
98*	Abortion usually generates depression in women who practice it	2.67	1.21	.165	-.752	.615	.655
100*	Women who have abortions do not usually maintain stable relationships	1.57	.98	1,732	2,433	.783	.775
108*	Women who abort with medications are often at risk in their future pregnancies	2.54	1.06	.084	-.199	.436	.516
Discrimination							
11	A woman who has had an abortion should not be judged	1.47	.980	2,316	4,837	.738	.720
13	A man would prefer to marry a woman who has never had an abortion	1.23	.660	3,180	10,887	.648	.760
15	I would not recommend you as a couple to a friend, a woman who has had an abortion	1.32	.820	2,999	9,458	.708	.768

16*	I would be disappointed if I knew that someone I love had an abortion	1.51	1.07	2,161	3,674	.743	.741
17	Women who abort should go to jail/be prosecuted for it	1.30	.830	3,134	9,786	.811	.866
25*	Women should be ashamed to share their decision to abort publicly	1.46	.910	2,398	5,923	.834	.820
27	A man should not forgive his partner if he knows that abortion has been performed	1.32	.780	2,620	6,575	.540	.639
28*	If a man finds out that his wife/partner had an abortion without his knowledge, he should report her	1.64	1.12	1,631	1,527	.785	.749
41*	I would try to dissuade a friend from having an abortion	2.17	1.37	.857	-.556	.700	.561
52	If I found out that a friend had an abortion, I would worry that she would be careful in her sexual relations	3.09	1.27	-.379	-.953	.378	.305
54	A responsible family should not allow a member of their family to have an abortion	1.47	.98	2,177	4,020	.884	.865
56	A woman who has decided to terminate her pregnancy does not deserve psychological support	1.23	.74	4,029	16,835	.524	.623
61	I think a woman should go to jail if she has an abortion, even if it's my friend or relative	1.36	.90	2,721	6,867	.848	.886
63	A woman who has an abortion should be treated the same as everyone else	1.47	.96	2,297	4,964	.561	.609
71	A woman who has had an abortion should not be in charge of my children's education	1.27	.70	2,916	9.04	.712	.812
80	I would rather not have to relate with women who have had an abortion	1.26	.70	3,133	10,509	.691	.818
86	If I found out that a friend had an abortion, our relationship would never be the same	1.27	.66	3,049	10,864	.727	.833
88	If I found out that a friend had a voluntary abortion, I would try to make her feel remorse	1.33	.75	2.44	5,958	.806	.852
89	A woman who has decided to terminate her pregnancy deserves to suffer	1.21	.63	3,448	13,454	.745	.873
96*	Understandably, a man rejects a woman for having had an abortion in the past	1.39	.90	2,545	6,198	.533	.605
99	A woman who has had an abortion does not deserve to be a mother or have other children	1.27	.72	3,248	11,742	.782	.892
102	A woman who aborts doesn't deserve pain medication	1.21	.70	3,896	15,996	.629	.735
103	A woman who has had an abortion should be prohibited from attending religious services	1.24	.65	3,151	11,113	.236	.280
106	If a relative aborted voluntarily, I would stop treating her like part of the family	1.20	.58	3,739	17.4	.717	.84
Morality							
7	Abortion is a no to life	1.97	1.44	1,239	-.021	0.805	.775
8	A woman who decides to have an abortion is committing an unforgivable sin	1.42	.96	2,464	5.58	.797	.793
12	Women who respect life do not abort	1.64	1.14	1,783	2,129	.786	.780
18	Not having premarital sex is the best solution to reduce abortion rates among young single women	1.42	.92	2,413	5,456	.662	.677
19	Abortion threatens the role of women in the family	1.39	.90	2,617	6,569	.696	.718
24	Abortion is always wrong	1.54	1.10	2013	2,954	.882	.891
26	A woman who decides to abort loses value as a person	1.25	.82	3,493	11,821	.758	.738
29	It is more understandable that a woman decides to abort if she has been raped, her life or that of the fetus is in danger, than if you decide to abort for another reason	2.37	1.39	.531	-1,134	.448	.421
39	Abortion is justifiable only in certain cases	1.98	1.23	1,089	.008	.468	.439
40	Abortion is against morality	1.52	1.08	2,112	3,442	.831	.820
43	God will punish women who practice abortion	1.45	.96	2.2	4,187	.746	.792
46	Abortion is justified only in cases of clear danger of death for the mother	1.64	1.08	1,696	1997	.723	.745
49	It is morally more reprehensible for a woman to have had two abortions than to have one	1.80	1.13	1,281	.65	.547	.557
53	Women were born to be mothers	1.42	.87	2,401	5,936	.667	.693
55*	Women who decide not to abort are blessed by God	1.53	1.00	1,878	2,761	.76	.812
58	Women who decide to abort reject their identity as women	1.39	.91	2,719	7,289	.639	.67
64*	Only God can take life	2.07	1.36	.905	-.462	.753	.765
72*	Women from birth have a maternal instinct	1.56	1.04	1936	2,945	.717	.723

74	Regardless of age, women are gifted at nurturing	1.42	.93	2.41	5.31	.706	.711
76	Women who abort should go to church to apologize for their actions	1.34	.83	2,861	8,543	.726	.716
79	Abortion is normal practice. It can happen to anyone	2.39	1.25	.492	-.719	.741	.707
81	Women who decide to abort are a source of shame for the family	1.24	.65	3,151	11,113	.733	.745
94*	Abortion is murder	1.70	1.29	1,569	.969	.870	.847
95	Terminating a pregnancy to save the life of the mother is not really an abortion	3.15	1.27	-.165	-.818	-.111	-.125
97	I reject abortion because I believe that life begins from conception	1.81	1.41	1,452	.495	.910	.899
105*	Getting to prevent a woman from interrupting her pregnancy is a source of pride	1.71	1.10	1,472	1,277	.580	.567

Note : * scale items selected to be part of the CAAS. *M* = Mean; *SD* = Standard deviation ; *Skew* = Skewness ; *Kurt* = Kurtosis ; *CTIC-CS* = Correlation corrected total item-Total; *CITC-TD* = Corrected item-total correlation- Theoretical Dimensions.

Annex 2

Descriptive statistics and corrected total item correlation (with complete scale and by theoric dimensions) of the CAAS in the study sample (N = 1223)

**Items**		**M**	**SD**	**Skew**	**Kurt**	**CTIC CS**	**CITC TD**
The prime of your life							
16	What a woman wants for her life cannot be truncated by an unwanted pregnancy	3.24	1.31	-.253	-.969	.440	.512
22	Termination of a pregnancy is justified if necessary for a prime life	3.19	1.26	-.171	-.923	.540	.552
24	If a woman has no desire to gestate and be a mother, you don't have to do it even if you get pregnant	3.41	1.28	-.327	-.929	.683	.565
Positive Stereotypes							
3	The decision to terminate a pregnancy is a sign of parental responsibility	3.24	1.37	-.301	-1.07	.379	.374
11	The woman who decides to abort has self-love for giving herself what she wants in life	3.13	1.22	-.126	-.749	.606	.596
17	Women who choose abortion are strong for defying the traditional mandate of motherhood	2.84	1.28	.089	-.958	.518	.693
25	A woman who aborts is a woman with the strength to go against what society expects of her	2.84	1.28	.100	-.951	.563	.723
32	Women who choose to abort are brave for challenging the status quo	2.74	1.25	.125	-.86	.533	.707
Right							
1	Women should not be required to discuss the abortion decision with others	3.12	1.51	-.163	-1,413	.287	.293
5	I believe that girls should have the right to abort	3.67	1.35	-.705	-.677	.729	.625
12	A woman has the right to abort as many times as necessary	2.97	1.44	.026	-1,303	.704	.576
19	Forcing a woman to carry an unwanted pregnancy to term should be understood as a violation of human rights	3.5	1.36	-.462	-.964	.537	.463
Negative Stereotypes							
2	Women who do not hesitate to abort are radical feminists	2.06	1.20	.898	-.165	.559	.555
8	A woman carries the trauma of abortion all her life	3.20	1.25	-.253	-.778	.466	.521
9	Many of the women who decide on abortion were not cautious enough to avoid finding themselves in this situation	2.56	1.37	.336	-1,125	.631	.651
10	A woman who aborts is a murderer	2.00	1.27	.961	-.300	.784	.702
14	Young women take abortion as a game	2.60	1.36	.269	-1.15	.746	.730
20	A girl should consider other alternatives to abortion to avoid trauma	2.92	1.22	-.129	-.878	.563	.568

23	The health of a woman who has an abortion is never as good as it was before the abortion	2.38	1.12	.226	-.772	.576	.631
28	Abortion usually generates depression in women who practice it	2.91	1.10	-.234	-.384	.557	.649
29	Women who have abortions do not usually maintain stable relationships	2.20	1.11	.426	-.699	.684	.706
31	Women who abort with medications are often at risk in their future pregnancies	2.86	1.03	-.235	-.138	.468	.539
Discrimination							
4	I would be disappointed if I knew that someone I love had an abortion	2.22	1.37	.745	-.752	.733	.706
6	Women should be ashamed to share their decision to abort publicly	1.89	1.15	1,126	.323	.657	.636
7	If a man finds out that his wife/partner had an abortion without his knowledge, he should report her	2.36	1.32	.517	-.916	.631	.630
13	I would try to dissuade a friend from having an abortion	2.71	1.38	0.160	-1,211	.565	.454
27	Understandably, a man rejects a woman for having had an abortion in the past	1.86	1.06	1,037	.226	.557	.564
Morality							
15	Women who decide not to abort are blessed by God	2.01	1.21	.868	-.320	.663	.711
18	Only God can take life	2.44	1.43	.450	-1,143	.677	.672
21	Women from birth have a maternal instinct	2.32	1.23	.455	-.877	.514	.514
26	Abortion is murder	2.25	1.38	.691	-.841	.815	.708
30	Getting to prevent a woman from interrupting her pregnancy is a source of pride	2.27	1.14	.411	-.719	.476	0488

Note : *M* = Mean; *SD* = Standard deviation; *Skew* = Skewness; *Kurt* = Kurtosis; *CTIC CS* = Correlation corrected total item-Total; *CITC-TD* = Corrected item-total correlation- Theoretical Dimensions.

Annex 3

Definitive items of the CASS in Spanish

N°	Ítem
**Autonomía**	
05	La mujer que decide abortar, tiene amor propio por darse a sí misma lo que quiere en la vida
06	Una mujer tiene derecho a abortar tantas veces como sean necesarias
09	Lo que una mujer quiere para su vida no puede verse truncado por un embarazo no deseado
10	Las mujeres que escogen abortar son fuertes por desafiar el mandato tradicional de la maternidad
12	Forzar a una mujer a llevar un embarazo no deseado a término debiese ser entendido como una violación de los derechos humanos
14	La interrupción de un embarazo está justificada, si es necesario para una vida plena
15	Si una mujer no tiene deseos de gestar y ser madre, no tiene por qué hacerlo, aunque se quede embarazada
16	Una mujer que aborta es una mujer con fortaleza por ir en contra de lo que la sociedad espera de ella
**Estigma**	
01	Me sentiría decepcionada/o si supiera que alguien a quién amo ha abortado voluntariamente
02	Las mujeres deberían avergonzarse de compartir públicamente su decisión de abortar
03	Muchas de las mujeres que acuden al aborto no fueron lo suficientemente precavidas para evitar encontrarse en esta situación
04	Una mujer que aborta es una asesina
07	Las mujeres jóvenes se toman el aborto como un juego
08	Las mujeres que deciden no abortar son bendecidas por Dios
11	Solo Dios puede quitar la vida
13	Las mujeres desde que nacen tienen instinto materno
17	Es entendible que un hombre rechace a una mujer por haberse practicado un aborto en el pasado
18	Las mujeres que se realizan abortos, no suelen mantener relaciones estables
